# Adenosine deaminase activity modulation by some street drug: molecular docking simulation and experimental investigation

**DOI:** 10.1186/2008-2231-22-42

**Published:** 2014-05-02

**Authors:** Massoud Amanlou, Ali-akbar Saboury, Roya Bazl, Mohammad Reza Ganjali, Shokoofeh Sheibani

**Affiliations:** 1 Department of Medicinal Chemistry, Faculty of Pharmacy, Pharmaceutical Sciences Research Center, Tehran University of Medical Sciences, Tehran, Iran; 2 Institute of Biochemistry and Biophysics, University of Tehran, Tehran, Iran; 3 Center of Excellence in Electrochemistry, Faculty of Chemistry, University of Tehran, Tehran, Iran

**Keywords:** Adenosine deaminase, Opioid, Cocaine, Immune system, Docking

## Abstract

**Background:**

Adenosine deaminase (ADA) is an enzyme that plays important roles in proliferation, maturation, function and development of the immune system. ADA activity may be altered by variety of substances including synthetic or natural products. Morphine, cocaine and their analogs exert immune suppressive activities by decreasing immune system function. The purpose of this study is to confirm that this possible effect may be modulated by interaction of these substances with ADA activity by experimental and computational method.

**Methods:**

The structural changes in ADA have been studied in presence of cocaine, ethylmorphine, homatropine, morphine and thebaine by determination of ADA hydrolytic activity, circular dichroism and fluorescence spectroscopy in different concentrations. Docking study was performed to evaluate interaction method of test compound with ADA active site using AutoDock4 software.

**Results:**

According to in-vitro studies all compounds inhibited ADA with different potencies, however thebaine activated it at concentration below 50 μM, ethylmorphine inhibited ADA at 35 μM. Moreover, fluorescence spectra patterns were differed from compounds based on structural resemblance which were very considerable for cocaine and homatropine.

**Conclusion:**

The results of this study confirms that opioids and some other stimulant drugs such as cocaine can alter immune function in illegal drug abusers. These findings may lead other investigators to develop a new class of ADA activators or inhibitors in the near future.

## Introduction

Adenosine deaminase (ADA) is an enzyme that irreversibly converts adenosine to inosine [1]. This enzyme exists in all human tissues, but the highest levels and activity are found in the lymphoid system such as lymph nodes, spleen, and thymus [2]. It is also essential for the proliferation, maturation and function of T lymphocyte cells. It is assumed that ADA plays a crucial role in development of the immune system, while its innate deficiency causes severe combined immunodeficiency (SCID) [3]. Moreover, ADA activity changes in a variety of other diseases including acquired immunodeficiency syndrome (AIDS), anemia, various lymphomas, tuberculosis, and leukemia [4,5]. On the other hand, ADA regulates the levels of endogenous adenosine which results in immune system suppression by inhibiting lymphoid or myeloid cells [6,7], including neutrophils [8], macrophages [9], lymphocytes [10,11] and platelets [12].

Two distinct isoenzymes of ADA, known as ADA_1_ and ADA_2_, are found in mammalian [13-15]; former has highest levels of activity in spleen, and thymus whereas latter is found in other parts of body [15]. As the most abundant type of white blood cells that responds to infection and attacks of foreign invaders, neutrophils might possess more than one type of adenosine receptor [16], and adenosine regulates neutrophil function in an opposing manner through the ligation of ADA_1_ (immunostimulatory) and ADA_2_ (immunosuppressive) receptors [17].

A number of ADA inhibitors with various degrees of potency have been reported [18]. In one study, immunosuppressive and anti-inflammatory effects of FR234938, as a non-nucleoside inhibitor of ADA, were investigated [19]. Moreover, deoxycoformycin, another ADA inhibitor, has been investigated in treatment of colon carcinoma cells [20,21] and hematological neoplasms [22]. By contrast, ibuprofen [23] and medazepam [24] effects on immune deficiency have been reported. This revealed that purine compound may act as ADA activator; but still more experiments are needed to confirm this finding.

Opioids have variety of clinical applications. Naltrexone in low dosage can suppress human ovarian cancer and provides novel non-toxic therapies for the treatment of this lethal neoplasia [25]. The idea that opioids suppress the immune system and reduce resistance to infections is not new [26]. Several studies on animals and humans have illustrated that opioids can exert immunosuppressive effects by interfering B and T cell function [27-29]. In this regard, Sacerdote et al. have reported that immune function is affected by morphine and tramadol [30]. In other investigations, chronic treatment with morphine has been shown to affect the function of T cells and reduce immunity by directly interacting with cells of the immune system [28,30-34]. On the other hand, studies about heroin abusers showed that patients were suffering from a disease that diminishes their immunity, by affecting T-lymphocyte function and therefore cause (HIV) infection [33,34]. In addition, long term usage of cocaine and homatropine leads to a heart attack, tremors, and apnea, cardiac arrest respectively. The possible involvement of adenosine in opioid antinociception has been supported by Ho et al. [34]. Interaction between an opioid and an adenosine receptor has been proposed. Yet, binding efficiency of ADA1 is reduced in the presence of morphine [35,36]. Also, transferring of opioids to the pontine reticular formation (PRF) and substantia innominata (SI) causes adenosine to decrease in the PRF and SI [37].

Although the knowledge about effects of opioids on immune responses has been improved, there is relatively little information about these immunosuppressive effects. Therefore, the present investigation was conducted to find the effects of this group of opiates and two other alkaloid compounds on ADA activity; by means of computational and experimental methods to found new compounds for modulation of adenosine deaminase activity.

## Material and methods

### Materials

Adenosine deaminase (EC 3.5.4.4) extracted from bovine spleen was purchased from sigma, and all other used materials were of analytical grade and acquired from Merck. PBS that is used in assay was adjusted at 7.4, at which, enzyme has optimal activity in [38,39]. All solutions were prepared in MilliQ (Millipore, USA) water.

### Biological assays

Released ammonia resulted by enzyme activity was determined by specific Berthelot colorimetric method which was used for micro determination of ADA activity in serum [40]. Briefly, this method is based on the reaction of liberated ammonia with hypochlorite (OCl^−^) to form a monochloramine and subsequent reaction of this intermediate with phenol to produce blue-colored indophenols that absorbance is measured at 625 nm. In this process sodium nitroprusside is used as the catalyst. The reaction was initiated by addition of adenosine as a substrate at 37°C to preincubated enzyme with different concentrations of tested compounds at different concentrations (5–300 μM). Finally, the enzymatic reaction was stopped at the end of 30 minutes of incubation by adding phenol nitroprusside solution. The mixture kept again for 30 minutes at 37°C before absorbance determination.

Obtained absorbance was normalized through blank sample, and IC_50_ values were calculated by Prism software (Ver. 5, GraphPad Software Inc., San Diego CA,).

### Circular dichroism spectroscopy

CD spectra were measured in the far-UV (200–260 nm) region with JASCO J-715 spectropolarimeter (Japan). JASCO J-715 software not only gave us the possibility of data smoothing, but also used to predict the secondary structure changes of the protein according to the statistical method [41]. Each scan was recorded in 1 nm increments at 37°C, repeated three times, and averaged. The protein was in 0.1 M PBS buffer, pH = 7.4, and its concentration was adjusted to 2 mg/ml in 1 cm path length cuvette. The results were expressed in molar ellipticity [θ] (deg cm^2^.dmol^−1^).

### Fluorescence spectroscopy

Fluorescence spectroscopy was performed on a Hitachi fluorescence spectrophotometer model MPF-4 with 1 cm path length fluorescence cuvette and final volume of 400 μL. The excitation wavelength was adjusted in 290 nm, and the emission was scanned every 1 nm in the range of 300 to 400 nm. The final concentration of enzyme was 6 μM and different concentrations of tested compounds were changed from 1.7 to 73.4 μM.

### Docking simulation

To have a better understanding about the inhibitory mechanism of the test compounds and clarification of type of interactions, docking were performed using the AutoDock 4.2 package [42]. The ability of software in next predictions was determined with re-docking of co-crystallized inhibitor of protein x-ray structure pdb ID: 1ADD and compared to inhibitor orientation in crystal structure. This crystal structure has been used since its > 80% similarity in structure with bovine spleen and active site amino acids conservation. The structure of all compound have sketched by Marvin sketch applet (Marvin package, Chemaxon Company). AutoDockTools (ADT) 1.5.6 was used for preparing input files using Autodock 4.2 atom types and calculating all needed charges [42]. Adding polar hydrogens and rotatable bonds were done with ADT; docking with a maximum number of 25 × 10^6^ energy evaluations using the Lamarckian Genetic Algorithm (LGA) were performed. All other parameters were set to default values. The docked conformations of each ligand were ranked into clusters based on the binding energy. After clustering analysis, conformation with the most favourable binding energy was selected.

## Results and discussion

### Determination of ADA activity

The target compounds were evaluated against ADA enzymatic activity in vitro and obtained IC_50_ values are summarized in Table 1. As shown in Table 1 all tested compounds inhibit ADA activity in micromolar range. In general, compounds in group1 (consisted of ethylmorphine, morphine and thebaine) showed better inhibitory activities than compounds in the other structurally related group (group 2: cocaine and homatropine). As shown, despite of partially closed binding energies, each compound inhibits the enzyme in variety of concentrations. Enhance in inhibition potency may be due to a decrease in conformational flexibility and better stabilization in inhibition site. In first group the order of inhibitory activities showed the potency of ethylmorphine > morphine > thebaine. This tendency is due to the presence of different substitute on A and C rings of perhydrophenanthrene structure. Ethylmorphine with ethoxy group on A ring shows better potency (IC_50_: 35 μM) than those with hydroxyl and methoxy substitutes. Upon further modification on C-ring in thebaine, the activity further decreased. This drop in inhibition activity may be due to inappropriate stabilization of compound to bind to target amino acids or inappropriate interactions which was confirmed by the molecular docking study. The binding energy of thebaine was calculated −7.2 kcal/mol compare to ethylmorphine (ΔG°: −8 kcal/mol). Interestingly in this series, thebaine activated enzyme hydrolytic activity till 50 μM and up to this level showed inhibition effect with IC_50_: 163 μM (Table 1). However, thebaine, cocaine and homatropine shows IC_50_ higher than other inhibitors (ethylmorphine and morphine), it can imply some immunity problem in the opioid consumers may result by ADA inhibition.

**Table 1:** **IC**_**50 **_**values and binding energies of test compounds in presence of 2 mg/ml adenosine deaminase enzyme**

**No.**	**Name**	**Structure**	**IC**_**50 **_**(μM)**	**ΔG° binding (kcal/mol)**
1	Thebaine		50*/163	−7.2
2	Morphine		43.7	−8.1
3	Ethylmorphine		35	−8.0
4	Cocaine		180	−6.8
5	Homatropine		96	−7.2

*Thebaine was activated ADA till this concentration.

In another group hydroxyl substitution may be responsible for the homatropine inhibition efficiency which might build H-bond with adjacent residues at active site of ADA. In addition, cocaine showed weaker inhibitory activity which indicated the influence of position and size of the substitute on tropan ring on the inhibitory activity. For more insight to obtained IC_50_ values and interaction site recognition the docking study has been performed.

### Docking experiments

Docking studies are used at different stages of drug discovery such as in the prediction of ligand-receptor interaction and also to rank the compounds based on the binding energies. Docking of co-crystallized inhibitor with ADA was performed to evaluate the efficacy of docking software and the reasonable RMSD of 0.39 Å was obtained. Docking of tested compounds with the ADA enzyme was performed, and corresponded binding energies were determined as shown in Table 1. The interacting energies followed the order of the in vitro IC_50_ values with rational correlation (R^2^: 0.84). All compounds posed in ADA active site entrance or partially penetrated in active site.

**Figure 1:**
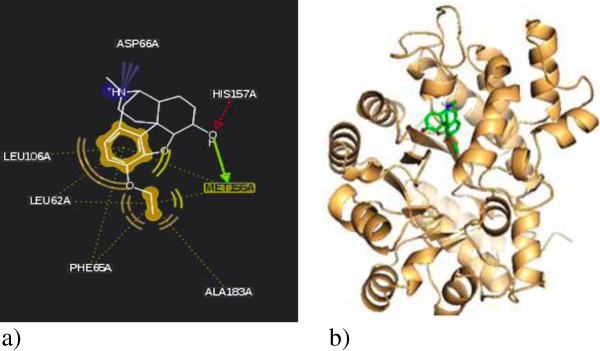
Ethylmorphine binding site of adenosine deaminase 2D (a) and 3D (b) demonstration.

**Figure 2:**
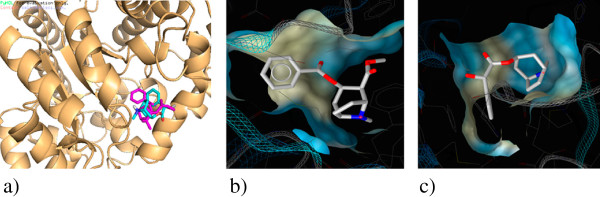
Docking pose of cocaine and homatropine in the adenosine deaminase binding pocket at their lowest energy of binding conformation: a) Overlay of cocaine and homatropine. b) Cocaine and c) Homatropine.

For the most potent inhibitor, ethylmorphine, the hydroxyl group of C-ring formed a hydrogen bond with Met155 and His157. Moreover, presence of ethoxy group in ring A resulted in hydrophobic interactions with Leu62, Phe65, Leu106, Met155 and Ala183 as well as electrostatic interaction of nitrogen atom made it much more stabilized in binding site (Figure 1). Thebaine exhibited binding energy of "-7" kcal/mol which correlated with the experimentally observed activity due to flat T-shape structure which resulted in less penetration into active site. On the other hand, in comparison to morphine replacing of hydroxy with methoxy group on C-ring caused removing of H-bond with His157, consequently resulted in lower efficiency. This effect was illustrated in ethylmorphine which in existing H-bond made it more stabilized in binding site. In the other group, homatropine binds to ADA with lower binding energy than cocaine with IC_50_ 96 and 180 μM respectively. As it shown in Figure 2 cocaine doesn’t diffuse into active site because of bulkier substitute, and just poses on enzyme active site entrance. In contrast, homatropine enters from phenyl part and OH group makes H-bond with Leu62.

### Fluorescence spectroscopy

**Figure 3:**
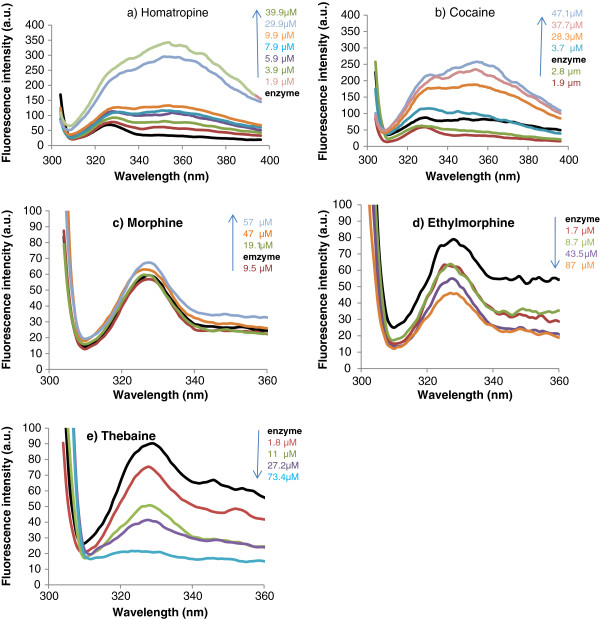
Change in fluorescence spectra of adenosine deaminase in presence of different concentration of a), Homatropine, b) Cocaine, c) Morphine, d) Ethylmorphine, and e) Thebaine in pH: 7.4.

Interaction of tested compounds with Trp residues can be studied by change in emission spectra of ADA in 320-340 nm. In other words, any environmental alteration as well as three-dimensional structure changes in Trp amino acid and enzyme respectively, can change the innate spectrum of Trp residues in enzyme. As shown in Figure 3, there are two totally different patterns in interaction with Trp residues. In the first group, homatropine and cocaine in high concentrations, demonstrate bathochromic shift. This change may be probably due to severe change of three dimensional structure of the enzyme. In the other series: morphine, ethylmorphine and thebaine do not show noticeable change and shift in fluorescence spectra; indeed compounds with bulkier structure don’t penetrate efficiently in binding pocket, and place in longer distance with Trp residues. Thebaine and ethylmorphine in this group decrease Trp emission as their concentration increase. This could be an indication that these compounds have bonded with the active sites or other sites of the ADA enzyme and therefore the Trp amino acid is out of access. According to the Berthelot test results, thebaine till 50 μM stimulates enzyme to break down faster, but above this concentration the activity of thebaine slows down and the concentration of 163 μM, results in 50% of inhibition.

### Circular dichroism spectroscopy

**Figure 4:**
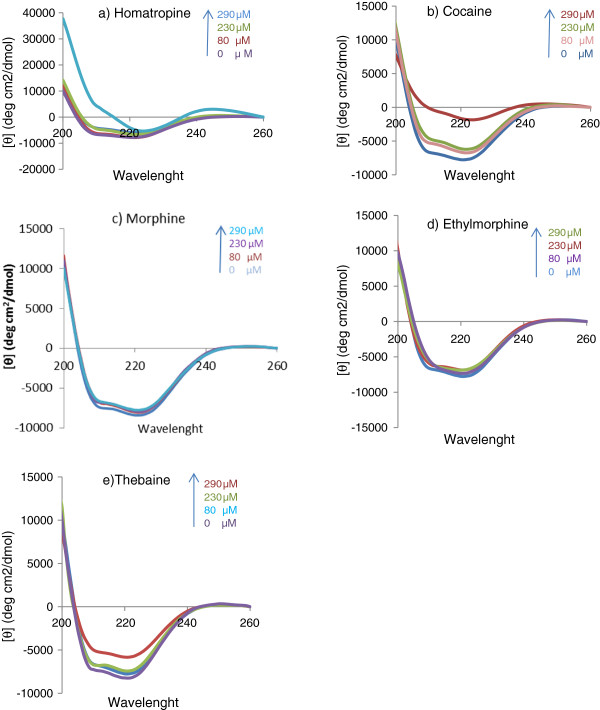
CD spectra of adenosine deaminase in presence of different concentration (0–300 μM) of a) Homatropine, b) Cocaine, c) Morphine, d) Ethylmorphine, e) Thebaine in pH: 7.4.

CD spectra of ADA in the presence of different concentration of compounds have been illustrated in Figure 4. ADA showed two transitions in 210 and 222 nm which are attribute to n → π* and π → π* respectively. According to percent of different patterns of 2D structure of enzyme, among α-helix structure has been undergone sever changes, and mostly convert to random coil. This effect can be explained by compounds intercalation between helix residues and broken of H-bonds. According to the Figure 3 in high concentrations of cocaine, bathochromic shift in the fluorescence spectra was observed. On the other hand as it shown in Figure 4, in cocaine concentration around 180 μM drastic changes in CD spectra were observed. Also, based on in-vitro studies 50% of enzyme’s activity has been diminished in 180 μM, so it can infer from this point despite of strong alteration in overall ADA structure or active site remains much more intact.

## Conclusion

Regarding to immunosuppressive effects of opioids, evaluation of enzyme activity studies was performed parallel to structural changes caused by different concentrations of test compounds. Thebaine activated ADA in certain low concentrations; while in higher concentrations it inhibited the enzyme. On the contrary, all other compounds inhibited the enzyme in studied range (0-300 μM). The interaction site as well as existing interactions in stabilization of each compound was obtained by docking studies. Finally, changes in two and three dimensional structures of ADA (based on Circular dichroism and fluorescence spectroscopy Figure 3 and 4) revealed that only cocaine and homatropine, had major effects on the enzyme (made drastic changes in the enzyme); while these changes were not significant for morphine, etylmorphine and thebaine in studied concentrations.

Regarding that the adenosine deaminase enzyme has a very important role in the immune system activity, so the inhibition of this enzyme's natural function in the human body can lead to debilitation of the immune system. Therefore, the results of this experiment support the idea that persons with addiction and regular drug abuse have a weaker immune system than others. This study can also help other researchers to develop a new class of ADA activators or inhibitors in the near future to manage malfunction of the immune system.

## Competing interest

There are no other conflicts of interest related to this publication.

## Authors’ contributions

All authors contributed to the concept and design, making and analysis of data, drafting, revising and final approval. MA is responsible for the study registration, financial and administrative support. RB & SHSH are responsible for biological assays. RB & MA were involved for docking studies. AAS & MRG were responsible for experimental analysis. All authors were participated in data assembly and analysis, interpretation and manuscript writing. They read and approved the final manuscript.
